# SEGCOND predicts putative transcriptional condensate-associated genomic regions by integrating multi-omics data

**DOI:** 10.1093/bioinformatics/btac742

**Published:** 2022-11-17

**Authors:** Antonios Klonizakis, Christoforos Nikolaou, Thomas Graf

**Affiliations:** Genome Biology Program, Centre for Genomic Regulation (CRG), The Barcelona Institute of Science and Technology (BIST), C/ del Dr. Aiguader 88, Barcelona 08003, Spain; Department of Medicine and Life Sciences, Universitat Pompeu Fabra (UPF), Doctor Aiguader 88, Barcelona 08003, Spain; Institute for Bioinnovation, Biomedical Sciences Research Centre ‘Alexander Fleming’, Fleming 34, Vari 16672, Greece; Genome Biology Program, Centre for Genomic Regulation (CRG), The Barcelona Institute of Science and Technology (BIST), C/ del Dr. Aiguader 88, Barcelona 08003, Spain; Department of Medicine and Life Sciences, Universitat Pompeu Fabra (UPF), Doctor Aiguader 88, Barcelona 08003, Spain

## Abstract

**Motivation:**

The compartmentalization of biochemical reactions, involved in the activation of gene expression in the eukaryotic nucleus, leads to the formation of membraneless bodies through liquid–liquid phase separation. These formations, called transcriptional condensates, appear to play important roles in gene regulation as they are assembled through the association of multiple enhancer regions in 3D genomic space. To date, we are still lacking efficient computational methodologies to identify the regions responsible for the formation of such condensates, based on genomic and conformational data.

**Results:**

In this work, we present SEGCOND, a computational framework aiming to highlight genomic regions involved in the formation of transcriptional condensates. SEGCOND is flexible in combining multiple genomic datasets related to enhancer activity and chromatin accessibility, to perform a genome segmentation. It then uses this segmentation for the detection of highly transcriptionally active regions of the genome. At a final step, and through the integration of Hi-C data, it identifies regions of putative transcriptional condensates (PTCs) as genomic domains where multiple enhancer elements coalesce in 3D space. SEGCOND identifies a subset of enhancer segments with increased transcriptional activity. PTCs are also found to significantly overlap highly interconnected enhancer elements and super enhancers obtained through two independent approaches. Application of SEGCOND on data from a well-defined system of B-cell to macrophage transdifferentiation leads to the identification of previously unreported genes with a likely role in the process.

**Availability and implementation:**

Source code and details for the implementation of SEGCOND is available at https://github.com/AntonisK95/SEGCOND

**Supplementary information:**

Supplementary data are available at *Bioinformatics* online.

## 1 Introduction

The finding that transcriptionally active regions in eukaryotic nuclei are spatially confined was first reported more than three decades ago (Jackson *et al.*, 1993). The concept of these structures, originally termed ‘transcription factories’ has been extended by the more recent discovery of transcriptional condensates within the eukaryotic nucleus that contain transcription factors and co-factors, such as MED1 and BRD4, as well as PolII and are associated with chromatin (Cramer, 2019). They have been proposed to materialize through liquid–liquid phase separation events into membraneless organelles that regulate the expression of key lineage genes (Hnisz *et al.*, 2017; Schoenfelder and Fraser, 2019; Stadhouders *et al.*, 2019). This process is likely dependent on weak interactions between low-complexity, intrinsically disordered domains (IDRs) of transcription factors and co-factors (Boija *et al.*, 2018).

Although the molecular mechanisms that drive the formation of transcriptional condensates are largely unknown, a subset of gene regulatory elements termed super-enhancers have been proposed to play a role in their assembly *in vivo* (Sabari *et al.*, 2018; Shrinivas *et al.*, 2019; Whyte *et al.*, 2013). Super-enhancers consist of hundreds of cell type-specific regions identified on the basis of exceptionally high occupancy of MED1, decoration with activation-related histone marks, such as H3K27ac and high density of transcription factor binding (Hnisz *et al.*, 2017; Whyte *et al.*, 2013). Super-enhancers have been described to drive high levels of lineage-specific gene expression (Whyte *et al.*, 2013) and proposed to function as scaffolds that concentrate transcription factors which subsequently lead to the formation of phase-separated structures (Blobel *et al.*, 2021). Evidence for this hypothesis has been provided for specific super-enhancer regions in mouse embryonic stem cells, including at the *Nanog*, *Trim28* and *Klf4* loci, coinciding with the detection of MED1 and BRD4 containing punctae in fixed cells (Sabari *et al.*, 2018).

The concept of super-enhancers has received some criticism (Blobel *et al.*, 2021; Hamdan and Johnsen, 2018; Moorthy *et al.*, 2017; Pott and Lieb, 2015). The currently used method for their identification, the ROSE algorithm (Lovén *et al.*, 2013; Whyte *et al.*, 2013), has some limitations as it only processes one type of ChIP-seq dataset at a time, it needs an *a priori* defined enhancer dataset and eventually stitches enhancers within a minimal distance into a new, larger enhancer. Evidence supporting the idea that large enhancer elements control cell-fate genes has been independently reported (Parker *et al.*, 2013). However, the notion that enhancer stretches serve as platforms that crowd transcription factors (Blobel *et al.*, 2021) has so far been tested only for super-enhancers and synthetic DNA (Schneider *et al.*, 2021; Trojanowski *et al.*, 2021). Thus, whether there are other genomic regions that can participate in the formation of condensates besides super-enhancers is not known.

In this report, we describe SEGCOND, a concise computational framework for the identification of potential transcriptional condensate-forming regions. Our method integrates several epigenetic and genomic parameters including histone marks, transcriptional regulator occupancy and chromatin accessibility. It also integrates conformational data (such as those obtained by Hi-C), to detect regions of increased potential to participate in condensates. Crucially, it implements a genome segmentation algorithm that is inspired from time-series forecasting models (Zeileis *et al.*, 2002, 2003), which allows greater flexibility in segment annotation than existing methods. We have developed SEGCOND using the data obtained with a time-resolved cell conversion system consisting of a B-cell line that can be transdifferentiated into macrophages (Borsari *et al.*, 2020; Choi *et al.*, 2021; Rapino *et al.*, 2013; Stik *et al.*, 2020). Functional analyses of potential condensate regions predicted by SEGCOND exhibit characteristics that overlap with super-enhancers and other genomic sequences, associated with high level expression of lineage-associated genes. These regions await validation by further experimental approaches.

## 2 Materials and methods

### 2.1 Datasets used

**Fig. 1.**
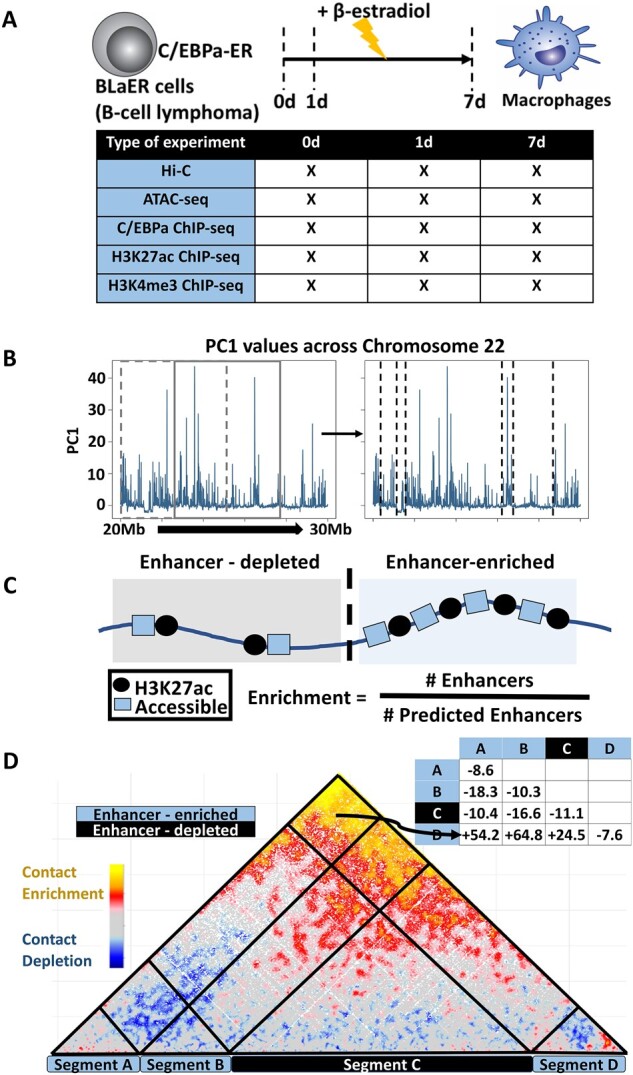
Datasets and description of methods used to develop the SEGCOND pipeline. (**A**) Diagram of the B-cell-to-macrophage transdifferentiation system and overview of the associated datasets used in this study. (**B**) Overview of the segmentation step. A genomic region in chromosome 22 at Day 0 is used as an example. Principal Component 1 values are calculated for 5 kb bins and are sorted across chromosomal coordinates. A sliding window of 1000 bins is used and a function aiming to detect break points is applied at each iteration. The window is slided 500 bins as depicted. The break points output is shown on the right. (**C**) Overview of the annotation step. Segments are classified as ‘enhancer-enriched’ or ‘enhancer-depleted’ based on a background zero-inflated negative binomial model that generates an ‘expected’ number of enhancers per segment. Annotation is performed on the basis of an observed over expected ratio. (**D**) Overview of the Hi-C integration step. SHAMAN normalized contact frequencies are pooled for intra/inter-segment interactions and the median values are reported in a symmetric matrix format

We used data obtained with a malignant human B-cell line to macrophage transdifferentiation system established in our lab (Rapino *et al.*, 2013). We integrated ATAC-seq, Hi-C and RNA-seq experiments from Stik *et al.* (2020), H3K27ac and H3K4me3 ChIP-seq experiments from Borsari *et al.* (2020) and C/EBPa ChIP-seq experiments from Choi *et al.* (2021). All datasets were available at three distinct timepoints during C/EBPa-induced transdifferentiation, namely 0, 1 and 7 days after induction (Fig. 1A).

### 2.2 Computational approach

Our goal was to develop a computational framework that enables the identification of candidate condensate-forming genomic regions through the integration and analysis of multi-omics data. Based on the identification of super-enhancers such regions are co-occupied by multiple transcription factors, display high nuclease accessibility and high levels of enhancer-related marks (Hnisz *et al.*, 2017; Sabari *et al.*, 2018). Condensate-associated regions have also been reported to harbour multiple 3D interactions between gene promoters and regulatory elements (Hnisz *et al.*, 2017; Sabari *et al.*, 2018).

The SEGCOND method consists of three distinct stages and is outlined below:

1. Omics-tracks integration and genome segmentation: We integrate multiple omics datasets and through dimensionality reduction and genome segmentation create a set of distinct genomic segments in linear chromosomes (Fig. 1B).
2. Segment annotation: Each segment is scored and assigned to a different functional class with the focus being on enhancer-associated properties (Fig. 1C).
3. Hi-C integration and candidate identification: 3D interaction between and within segments is scored with the integration of Hi-C data. Candidate regions are identified through the application of a set of thresholds associated with chromosomal interaction values (Fig. 1D).

#### 2.2.1 Omics-tracks integration and genome segmentation

As discussed above, condensate forming regions are expected to exhibit particular ‘footprints’ in 1D next-generation sequencing (NGS) data, related to chromatin accessibility, such as ATAC-seq, and gene regulation, such as ChIP-seq data of transcription factors and co-factors. In line with this assumption, the first step of the pipeline we generated is a genome segmentation process, in which genomic coordinates are broken up into segments based on the combined input of multiple omics datasets measuring protein occupancy, chromatin accessibility or any other 1D feature of the genome.

The desired input dataset in our analysis is multi-dimensional, as multiple different NGS datasets, such as ATAC-seq and ChIP-seq of different TFs, are to be used as input. To this end, we developed a custom segmentation algorithm, inspired from time-series data analysis (Fig. 1B). We treat chromosomal coordinates as the pseudo-time variable and proceed by

- Binning the genome in 5-kb bins, a bin size that is large enough to allow for the integration of Hi-C data.
- Normalizing the NGS read input per bin, using deeptools2 (Ramírez *et al.*, 2016).
- Applying a dimensionality reduction technique to ‘project’ data from multiple experiments into a 1D value. In our case, we performed a principal component analysis (PCA) analysis keeping the first principal component values (PC1) as they appear to capture a significant portion of the variance in our datasets (55.69%, 59.01%, 57.92% for timepoints 0, 1 and 7 days, respectively). Moreover, all datasets correlated positively with PC1 values and contributed similarly toward PC1 values, with the exception of C/EBPa ChIP-seq data (Supplementary Fig. S1A and B).
- Identifying boundaries in the 1D signal through the implementation of the R function breakpoints() from the strucchange package (Zeileis *et al.*, 2002). breakpoints() applies multiple linear regression models along the serial data and uses an *F*-test (Chow test) to identify boundaries between consecutive segments (Fig. 1B).

We should note here that as strucchange cannot process all of the values in a chromosome simultaneously, the algorithm is implemented via a sliding window approach. Windows are overlapping and identified boundaries that fall within the same window are merged. A window size of 1000 bins, corresponding to 5 Mb of DNA, was chosen after different lengths (250–1000 bins) provided robust and highly similar outputs. A final important parameter of strucchange is a given minimum segment size value, expressed as a percentage of the window size. No segments beneath this value can be returned by a single iteration of the algorithm. This value is important as it also imposes a threshold on the maximum structural breaks that can be calculated per iteration. We tested multiple cut-off values (1%, 2.5%, 5%, 7.5%, 10%, 15%, 20% and 25%) on the 0-day dataset. We evaluated the size of the generated segments (Supplementary Fig. S1C), the difference between the average PC1 values of segments with their neighbouring segments (Supplementary Fig. S1D) and the standard deviation of the PC1 values within segments (Supplementary Fig. S1E). We opted for a cut-off where segments are sufficiently large and show optimal behavior for the other two metrics we examined. We picked a cut-off of 5%, corresponding to 250 kb of DNA, for all further analyses.

#### 2.2.2 Segment annotation

In order to isolate segments that show an abundance of enhancer-associated features (Fig. 1C), we employed an annotation scheme based on a background zero-inflated negative binomial distribution. For each segment, we calculated the number of bins that simultaneously showed signature enhancer marks as high ATAC-seq and H3K27ac signals. To do so, we log10-transformed the initial data matrix and *Z*-score transformed the values of each experiment. A *Z*-score of ≥1, corresponding approximately to the top 5% of values, was imposed as a cutoff. Bins that had simultaneously *Z*-scores of ≥1 for both H3K27ac ChIP-seq and ATAC-seq samples were converted into values of ‘1’ while the rest to ‘0’ values. We aggregated the score of every segment.

In order to statistically evaluate which regions show an enrichment of enhancer bins, a background model was generated for every segment. Segments of equal size were randomly shuffled across the genome 1000 times and the sum of enhancer-bins was calculated for every random iteration. The random values were used to fit a zero-inflated negative binomial background distribution. A *P*-value and an enrichment score for each segment were obtained using this background distribution. Segments that had a *P*-value of less than 0.05 and a positive enrichment score were deemed as ‘enhancer-enriched’ segments. Other possible models were also fit to a series of randomly generated distributions: Negative Binomial, Normal and a Tobit-Normal model with negative values censored. We used Akaike’s information criterion to evaluate the performance of each model. In all cases tested, the Zero-Inflated Negative Binomial model proved to be performing better (Supplementary Fig. S2A and B).

#### 2.2.3 Hi-C integration and candidate identification

A Hi-C normalization algorithm, SHAMAN (Mendelson Cohen *et al.*, 2017), was used to generate normalized contact maps. SHAMAN was chosen as it provides flexibility regarding the integration of Hi-C data, given that it doesn’t require a predefined binning resolution for its operation. To create normalized SHAMAN Hi-C tracks per timepoint, we ran SHAMAN on our Hi-C data following SHAMAN’s documentation (https://tanaylab.bitbucket.io/shaman/articles/import.html and https://tanaylab.bitbucket.io/shaman/articles/shaman-package.html). Only filtered Hi-C reads were used, as described in Stik *et al.* (2020).

For every segment pair, the complete set of normalized interactions was pooled and the median value was calculated. Each segment is also assigned an intra-segment interaction score, based on the normalized interactions found within its own coordinates. For interaction scores between different segments, only segments within 2 Mb of each other were scored. This is due to the decaying number of SHAMAN normalized contacts (Supplementary Fig. S3A).

In order to identify ‘enhancer-enriched’ segments that exhibit a high intra-segment or inter-segment interaction score, a cut-off was estimated on the basis of a permutation test. Segment coordinates were shuffled randomly 100 times and Hi-C interaction scores were calculated for all pairwise random segment combinations. For a series of cutoffs, we tried to maximize the number of ‘true’ segment pairs passing the threshold minus the one of randomized segment pairs passing the threshold. The resulting number was averaged across permutations for each timepoint. A maximum was obtained at a cutoff of 17 after averaging the scores across timepoints as well (Supplementary Fig. S3B). Segments forming putative condensates are isolated after converting the SHAMAN interaction matrices into binary ones. An entry is transformed into a value of 1 (and thus considered to be linking segments in 3D) if it combined:

- A SHAMAN score ≥17.
- Both segments being ‘enhancer-enriched’.
- A distance of segments ≤2 Mb.

At the last step, the binary matrix was converted to a graph via the R igraph package (https://igraph.org/r/). Connected components were isolated and were labeled as putative condensates.

### 2.3 Benchmarking with ChromHMM

We benchmarked our method’s segmentation and annotation steps against a widely used segmentation algorithm, ChromHMM (Ernst and Kellis, 2012). (For a detailed description and discussion related to benchmarking. see ‘Supplementary Materials and Methods’ and Supplementary Figs S4 and S5). In brief, we observe a high degree of overlap between enhancer-related segments produced by SEGCOND and enhancer-related ChromHMM segments. However, ChromHMM identifies multiple additional segments compared with SEGCOND (up to two times more in certain cases) that are overall smaller in size, making the integration of Hi-C data impractical.

## 3 Results

### 3.1 Identification of putative transcriptional condensates

**Fig. 2.**
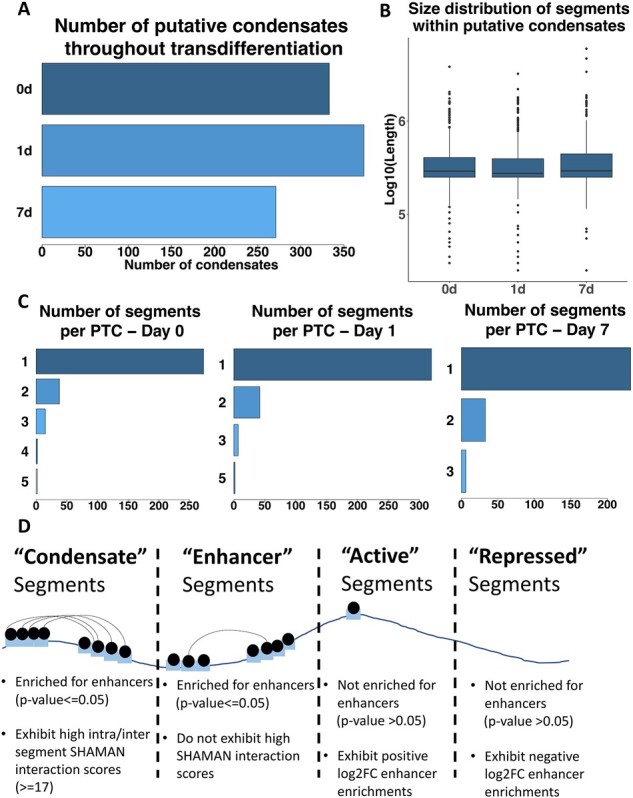
Number of identified putative condensates and segments found within. (**A**) Number of putative condensates per timepoint. (**B**) Size distribution of segments within putative condensates. No significant differences found between timepoints. (**C**) Distribution of number of segments comprising putative condensates per timepoint. (**D**) Separation of segments in four categories for downstream analyses

We employed SECGOND in datasets derived from three distinct timepoints of our B-cell to macrophage transdifferentiation system, to identify a set of regions that are enriched for enhancer-related marks and form strongly associating hubs in 3D space. We termed these regions as putative transcriptional condensates (PTCs). The number of PTCs identified ranged between 271 and 373 per sample at a given time point, showing that at the intermediate time point (Day1 of trans-differentiation) there were more PTCs than in either B cells (Day0) or induced macrophages (Day7) (Fig. 2A). The isolated hubs consisted of one to five segments each and were of comparable size in all three timepoints (Fig. 2B and C). We also checked the overlap of PTCs between different timepoints. 252 Day0 PTCs overlapped a Day1 PTC, while 181 Day0 PTCs overlapped a Day7 PTC. Finally, 204 Day1 PTCs overlapped a Day7 PTC. Overall, a big subset of PTCs per timepoint appeared to remain stable throughout transdifferentiation.

### 3.2 Candidate condensate segments identified by SEGCOND partially overlap with super-enhancers and highly interconnected enhancer communities

To our knowledge, SEGCOND is the first method that attempts to identify putative condensate regions. Due to the lack of other methodologies against which we could benchmark, we compared our PTCs with super-enhancer predictions using the ROSE algorithm and a set of highly interconnected enhancers (HICE), identified in an independent study (Madsen *et al.*, 2020). We ran ROSE (https://github.com/stjude/ROSE) with an input of enhancer regions (in our case, the overlap of H3K27ac + ATAC-seq peaks that were called with MACS2 for our data), using the default stitching parameter and distance parameters (12.5 and 2.5 kb, respectively). We then identified the genes lying within regions overlapping both our PTCs and ROSE’s super-enhancer predictions and found that roughly one out of five super-enhancer genes overlapped with PTCs (17%, 22% and 19% for Day 0, Day 1 and Day 7, respectively). More importantly, we found significant (almost 2-fold) higher than average expression of super-enhancer genes that overlapped our PTCs compared with non-PTC-associated super-enhancer (i.e. ROSE) genes. Finally, PTC-specific genes found in Day 0 and Day 1 also exhibited statistically significant higher expression compared with super-enhancer specific genes on the same timepoints, suggesting that our method is likewise able to detect regions that are enriched in transcriptional activity to a degree that is superior to the one that is pertinent to super-enhancers in general (Supplementary Fig. S6A).

In order to compare SEGCOND-identified PTCs with HICE elements, we run SEGCOND on an independent set of data described for adipocyte differentiation (Madsen *et al.*, 2020). We integrated MED1, C/EBPb, H3K27Ac and DNaseI data for the genome segmentation step and Hi-C for the definition of PTCs. We then tested the overlap of our defined PTCs with the HICE elements identified by the authors using a permutation analysis and found a highly significant colocalization, exceeding the one found for other actively transcribed elements (Supplementary Fig. S6B).

We conclude that SEGCOND is able to identify highly active enhancer regions with increased connectivity in 3D space. The elevated transcriptional activity of genes lying in the proximity of this enhancer subset further supports the notion that these regions exhibit properties of transcriptional condensates. The significant overlap of PTCs with super-enhancers as well as with HICE elements, even though the latter are not considered to be bonafide condensate-forming regions, is an additional indication of our method’s potential to combine linear genomic and 3D conformational data in a meaningful way.

### 3.3 Genes in putative condensates reflect transdifferentiation dynamics

**Fig. 3.**
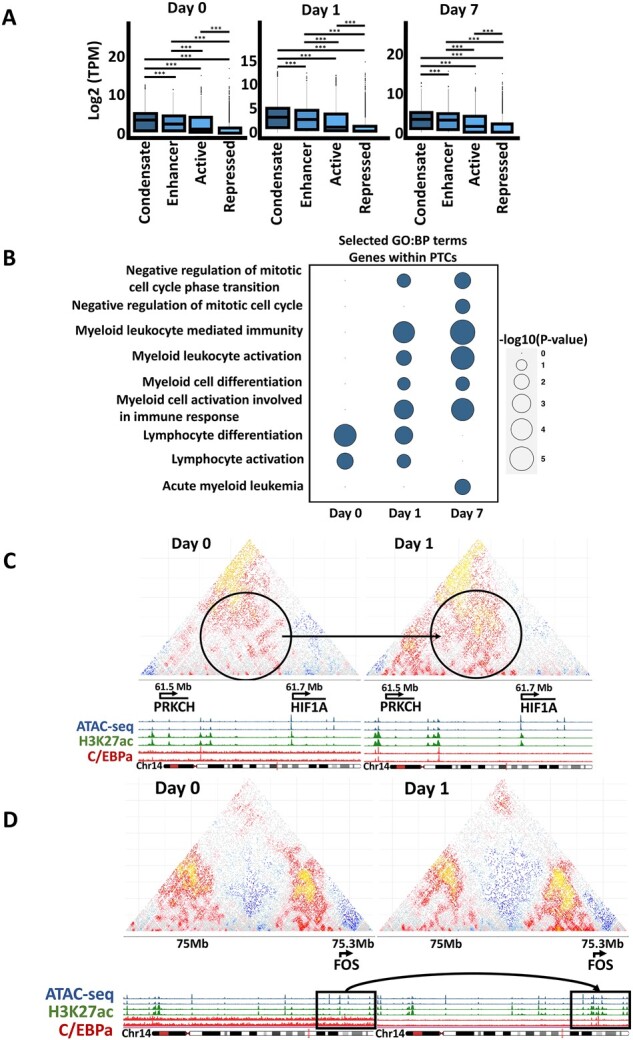
Functional characterization of putative condensate regions. (**A**) Expression of genes (as TPM) belonging to different segment types. Stars denote a Wilcoxon Rank Sum test *P*-value of less than 0.001. (**B**) Selected, enriched, GO: BP terms of genes residing in putative condensates per timepoint. Enrichment analysis was performed with gprofiler2. (**C**) SHAMAN Hi-C profiles showing changes in a putative condensate forming region during transdifferentiation within the Hif1a locus. This region is predicted not to be part of a condensate at Day 0 but at Day 1. Red and yellow pixels depict an enrichment of contact frequencies, while gray and blue depict no enrichment or depletion respectively. Note increased interaction frequencies within the circled region at Day 1. (**D**) SHAMAN Hi-C profiles showing changes in a putative condensate forming region of *Fos*. Note an increase in the H3K27ac signal at Day 1 within the highlighted square

Current literature suggests that transcriptional condensates preferentially control the expression of highly expressed lineage instructive genes (Sabari *et al.*, 2018). We thus focused first on the properties of genes contained in our putative condensate regions. We split our segments into four categories: ‘Condensate’ segments, ‘Enhancer’ segments, ‘Active’ segments and ‘Repressed’ segments (Fig. 2D). We identified the genes falling exclusively within the different segment types and calculated their transcript per million (TPM) values. These were plotted separately for each timepoint (Fig. 3A). Consistent with expectations, genes falling in ‘Condensate’ segments were significantly over-expressed compared with genes in genomic segments defined as transcriptionally active or enhancer containing.

Since our Day 0 B cells correspond to highly proliferating B-cell lymphoma cells and induced macrophages stop dividing, we used gProfiler2 (Kolberg *et al.*, 2020) to perform a GO-term enrichment analysis of genes falling within ‘Condensate’ segments at the three cell stages (Fig. 3B) to inquire for functional changes reflecting the dynamics. This showed that cells at Day 0 are enriched for lymphocyte related but not macrophage-related terms, while Day 7 cells are enriched for myeloid terms. Terms associated with cell-cycle arrest appear at Day 1 and persist until Day 7, consistent with the observation that C/EBPa induces a cell-cycle arrest (Rapino *et al.*, 2013). Moreover, Day 1 cells resemble an intermediate state, as both macrophage- and lymphocyte-related functions are enriched, consistent with recent reports (Borsari *et al.*, 2020).

Together, these results strongly suggest that our method detects regions with increased gene expression, which are functionally associated with the B-cells-to-macrophage transdifferentiation process.

### 3.4 SEGCOND captures putative condensate regions as potential new players in the transdifferentiation process

To search for candidate condensate regions that participate in transdifferentiation we focused on genes within PTC regions. Across the three timepoints, we identified 3849 genes that (i) were located within a PTC, (ii) had non-zero expression (TPM > 0) and (iii) had a promoter proximal region (±5 kb) accessible and decorated with H3K27ac. Out of these, 1021 were in PTCs consistently across the whole process. Of these, we identified 256 genes lying consistently within Day 1–Day 7-specific PTCs (but not Day0) and therefore can be considered potentially important for transdifferentiation. An example of such a candidate is the *Hif1a* gene (Fig. 3C). The *Hif1a* locus exhibits higher interaction scores at Day 1 than at Day 0, accompanied by a more than 2-fold increase in the gene’s expression levels, suggesting a functional role for the gene. An additional example is the *Fos* gene locus (Fig. 3D) where H3K27ac signal changes dramatically from Day 0 to Day1, alongside its expression levels (>60-fold increase). Candidate loci like these allow the experimental validation of novel genes that have so far not been implicated in the B-cell-to-macrophage cell fate conversion.

## 4 Discussion

Transcriptional condensates play key roles in processes ranging from transcription to translation, metabolism and signaling, however, there is a lack of existing methodologies for their identification from genomic readouts. The computational framework described here was developed to identify genomic regions that may act as nucleation points for the assembly of transcriptional condensates. SEGCOND’s segmentation approach is different from existing methods, mostly depending on Hidden Markov Models, by integrating Hi-C data. Existing HMM segmentation approaches lead to a fragmentation of genomic space that is incompatible with the resolution of current genome conformational data, such as Hi-C, which are, nonetheless crucial for condensate identification. Another major difference of SEGCOND from existing segmentation methods is the statistical approach, used for the annotation of the defined segments. This combines a permutation test and the use of a zero-inflated negative binomial distribution to assess the significance of each segment’s attribution to a given status. SEGCOND is also flexible in the possibility of incorporating additional data. Segment annotation is conditional to the original input and thus, it can identify both active and repressive regions depending on the combination of the genomics data used in the segmentation step.

As with all segmentation methodologies, one limitation of SEGCOND is the choice of parameters. Depending on the value used to identify structural breakpoints the resulting segments vary in size. Nevertheless, the median segment size does not vary more than half an order of magnitude even for a very extreme range of breakpoint parameters (see Supplementary Fig. S1C). Greater variability is observed, as would be expected, in the quantitative signal of the segments depending on their size, with smaller segments, corresponding to a larger fragmentation, having more intense signal changes (Supplementary Fig. S1D). Most of this variability is resolved through SEGCOND’s dimensionality reduction step, which distributes the variation more evenly across the breakpoint cut-off range (Supplementary Fig. S1E). This robust behavior allows the user to opt for different cutoffs and segment sizes without undermining the classification potential. The empirically chosen 5% cutoff guarantees an optimal trade-off between signal variability and a segment size distribution that reflects the expected length scale of the phenomenon under study.

As data accumulate and our knowledge on the mechanisms underlying condensate formation becomes enriched, new transcriptional regulators may be associated with the phenomenon. Integrating multiple TF ChIPSeq tracks via SEGCOND may require some additional care. Depending on the nature of the analyzed TFs, the user may opt to include all tracks in a single run, if they are expected to co-operate (and likely to co-localize). In case they are likely to exert complementary functions, multiple runs of SEGCOND would probably be more suitable, followed by a merging of the predicted candidate PTCs, each labeled under the TF from which they were derived.

Additional limitations of SEGCOND are related to computational demands. The application of the breakpoint function is memory demanding and thus needs to be run serially on sliding windows. This means that the method is only ‘aware’ of a section of the data each time. The Hi-C integration is also computationally intensive, especially for small segment sizes, which is imposed by the use of SHAMAN. Therefore, in its current version, SEGCOND requires the computational capacity of a computer cluster in order to perform the full set of necessary functions.

Besides the increased computational demands, our analyses show that SEGCOND is an efficient method for condensate prediction. The regions it proposes partially match a subset of super-enhancers and HICE hubs that are identified by two independent methodologies. We furthermore show that the identified PTCs harbor a subset of highly expressed genes, with expression levels that exceed all other genomic transcriptionally active regions. Application of SEGCOND to data from a well-studied transdifferentiation system allowed us to propose novel genes potentially involved in cell fate changes, testable in validation experiments. For example, DNA and RNA FISH experiments involving the identified regions could reveal whether they colocalize with MED1 punctae, as has been shown for super-enhancer regions (Sabari *et al.*, 2018). The link between specific enhancer elements in PTCs and the formation of transcriptional condensates could also be investigated using CRISPR-Cas9. To our knowledge, the computational method described here is the first one specifically designed toward proposing genomic regions participating in the formation of transcriptional condensates.

## Supplementary Material

btac742_Supplementary_Data
