# Supplementary material for: SEGCOND predicts putative transcriptional condensate-associated genomic regions by integrating multi-omics data
Source: Bioinformatics. 2022 Nov 17;39(1):btac742. doi: 10.1093/bioinformatics/btac742 (PMC9805567; doi:10.1093/bioinformatics/btac742)
Supplement: btac742_Supplementary_Data [file btac742_supplementary_data.pdf]

Supplementary Material

SEGCOND predicts putative transcriptional condensate-associated genomic regions by integrating multi-omics data

Antonios Klonizakis[1,2], Christoforos Nikolaou[3,*] and Thomas Graf [1,2,*]

[1]Centre for Genomic Regulation, Carrer del Dr. Aiguader 88, Barcelona

[2]Universitat Pompeu Fabra, Carrer de Ramon Trias Fargas, 25, 27, Barcelona

[3]Institute for Bioinnovation, Biomedical Sciences Research Centre "Alexander Fleming," 16672 Vari, Greece

*To whom correspondence should be addressed

Supplementary Materials and Methods

Benchmarking with ChromHMM

Well-established segmentation and genome annotation (SAGA) algorithms, such as ChromHMM (Ernst et al., 2012) employ Hidden Markov Models (HMM) to separate the genome into segments based on different combinations of histone marks, transcription factors binding and transcriptome profiles. Such approaches don't consider the actual values of the different "signals" as they binarize them into either "present" or "absent". Moreover, ChromHMM tends to over-fragment the genome (See Figure S4B). However, for genomic applications involving Hi-C data larger segments are more desirable, since their resolution, even for read-rich experiments, is not high enough to infer conclusions for small genomic regions (such as for regions of <200bp).

In spite of these limitations, and the lack of methodologies aiming at the length scale of interest, we chose ChromHMM to benchmark our method against. ChromHMM was run on the same set of data with different binning sizes. The sizes ranged from 1kb to 250kb and ChromHMM binary matrices were generated for each binsize by using the ChromHMM BinarizeBam (BAM files of ATAC-seq, H3K27ac and H3K4me3 ChIP-seq) and BinarizeBed -peaks (MACS2 narrowPeaks of C/EBPa ChIP-seq) commands. For every run, a set of 6 chromatin states were used. Six states were used as they resulted in emission matrices that were not redundant: That is that the final model states were not similar with one another as in case for models with more states. Models for different binsizes were generated with the Learn Model ChromHMM command. To determine ChromHMM states that were associated with enhancer-related marks, a PCA analysis was carried out for all emission matrices. After plotting the first two principal components, enhancer-like states clustered together and were isolated as they had a PC1 value of <-2.5 (Figures S4A). Any ChromHMM segment belonging to an enhancer-like state was termed as a "ChromHMM enhancer segment".

ChromHMM returned a larger number of enhancer segments than SEGCOND, irrespectively of the binning size chosen. In addition, the size of these elements was too

short to make integration of the Hi-C data feasible. We next evaluated the genes that overlapped between the ChromHMM generated regions and the Enhancer – Enriched segments generated by our approach. It can be seen that both methods are in high agreement but with our method returning a smaller set of genes (Figure S5A).

Transcriptional condensates have been associated with high expression of the target genes (Hnisz et al., 2017; Sabari et al., 2018). We therefore reasoned that Enhancer – Enriched segments should harbor highly expressed genes. Thus, for each Enhancer – Enriched segment, we isolated the expression levels of the genes found within and calculated the average value. We repeated the same analysis for ChromHMM enhancer-related segments. This showed that the average expression in the regions identified by our approach tend to be higher than with ChromHMM (Figure S5B). We also performed a permutation analysis to validate that Enhancer – Enriched segments harbor more expressed genes than ChromHMM segments. For each Enhancer – Enriched and ChromHMM enhancer segment, 100 random segments of equal size were generated using bedtools shuffle. For each of the random segments the average TPM of genes lying inside was calculated and thus a background distribution was generated.
An enrichment score was finally calculated for each segment:
Enrichment of segment$_i$ = log2 (Average TPM of Segment$_i$)/(Average of background distribution$_i$)

The enrichment scores are depicted in the form of boxplots (Figure S5C). Once again, SEGCOND Enhancer-related segments seem to harbor genes that are slightly higher expressed than ChromHMM enhancer segments.

Annotation of segments via ChromHMM

We also examined whether annotating SEGCOND segments with ChromHMM could be superior to our zero-inflated negative binomial background approach. To do so, we utilized the 5kb ChromHMM Enhancer-Associated segments that were described above. We chose a 5kb resolution as it matched the binsize that we used for the generation of SEGCOND segments. We calculated a simple enrichment score for each segment:

1. For each SEGCOND segment, we calculated the number of base pairs overlapping ChromHMM enhancer segments.

2. 5kb ChromHMM enhancer segments were randomly shuffled using bedtools shuffle. For every iteration, the number of base pairs overlapping SEGCOND segments was reported. This procedure generated a background random distribution for each SEGCOND segment,

3. A bootstrap p-value was calculated for each SEGCOND segment as:
   # of random iterations with equal or higher base pair overlap / 1000

As can be seen in Figure S5D, our zero-inflated negative binomial background model leads to a more refined subset of segments overall.

Supplementary Figures and Figure legends

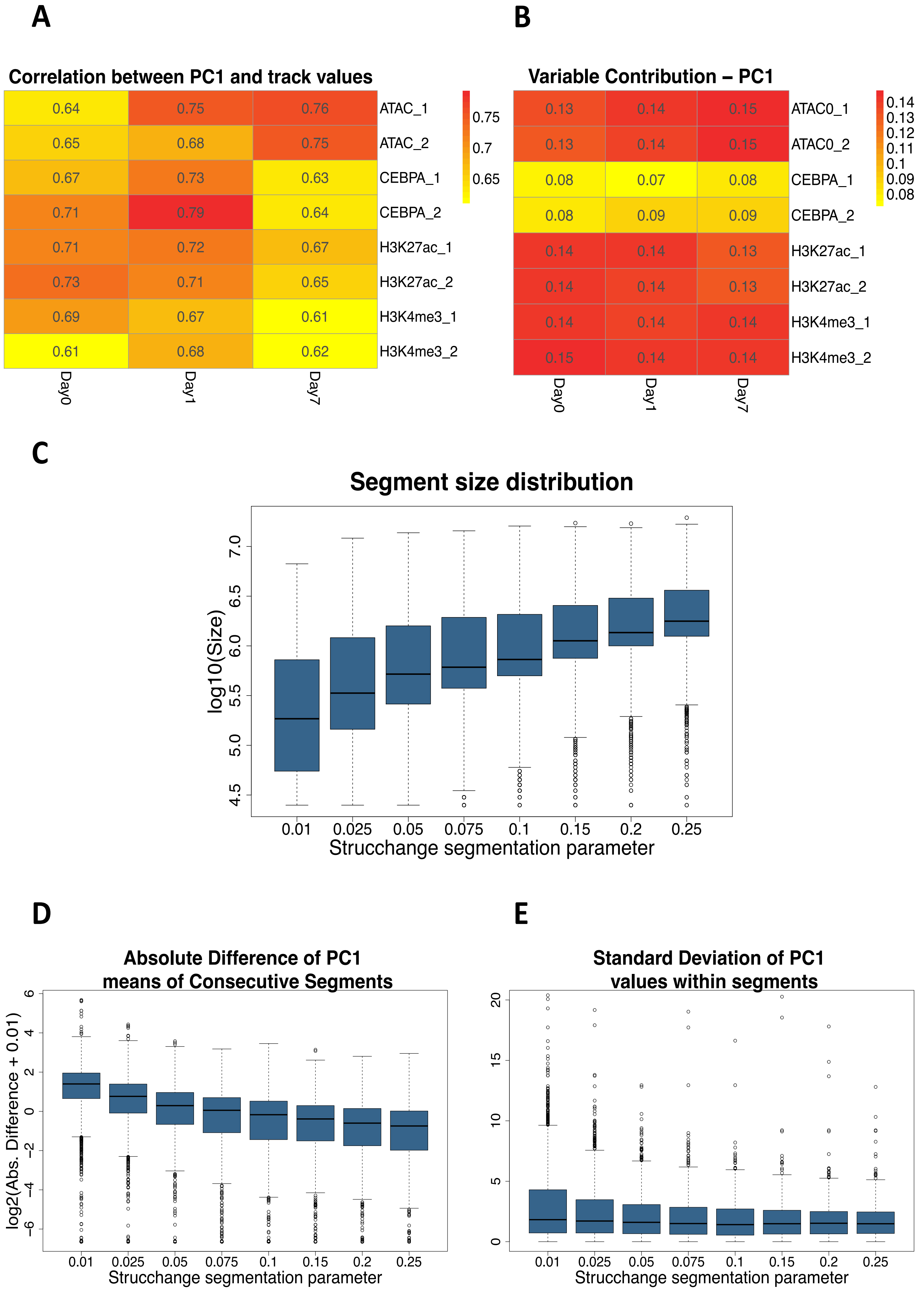
A
Correlation between PC1 and track values
0.64 0.75 0.76 ATAC_1
0.65 0.68 0.75 ATAC_2
0.67 0.73 0.63 CEBPA_1
0.71 0.79 0.64 CEBPA_2
0.71 0.72 0.67 H3K27ac_1
0.73 0.71 0.65 H3K27ac_2
0.69 0.67 0.61 H3K4me3_1
0.61 0.68 0.62 H3K4me3_2
Day0 Day1 Day7
0.75
0.7
0.65
B
Variable Contribution – PC1
0.13 0.14 0.15 ATAC0_1
0.13 0.14 0.15 ATAC0_2
0.08 0.07 0.08 CEBPA_1
0.08 0.09 0.09 CEBPA_2
0.14 0.14 0.13 H3K27ac_1
0.14 0.14 0.13 H3K27ac_2
0.14 0.14 0.14 H3K4me3_1
0.15 0.14 0.14 H3K4me3_2
Day0 Day1 Day7
0.14
0.13
0.12
0.11
0.1
0.09
0.08
C
Segment size distribution
log10(Size)
4.5
5.0
5.5
6.0
6.5
7.0
0.01 0.025 0.05 0.075 0.1 0.15 0.2 0.25
Strucchange segmentation parameter
D
Absolute Difference of PC1
means of Consecutive Segments
log2(Abs. Difference + 0.01)
−6
−4
−2
0
2
4
6
0.01 0.025 0.05 0.075 0.1 0.15 0.2 0.25
Strucchange segmentation parameter
E
Standard Deviation of PC1
values within segments
0
5
10
15
20
0.01 0.025 0.05 0.075 0.1 0.15 0.2 0.25
Strucchange segmentation parameter

Figure S1. Choosing a strucchange() segmentation parameter.

A: Spearman correlation coefficients between PC1 values and read-depth normalized values of each dataset per bin and per timepoint.

B: PC1 contribution of different datasets. After PCA was performed loadings of each variable were isolated. Afterwards, the overall contribution of each variable was calculated as:

|PCA loading| / Sum(|PCA loadings|)

C: Size distribution of generated segments for different strucchange() segmentation cutoff values.

D: Absolute difference between the PC1 means of consecutive segments. Since the genome is segmented based on PC1 values, the desired outcome is to have large differences between neighboring segments.

E: Standard deviation of PC1 values within segments called for different parameters. More homogeneous values are desired within segments, thus lower sd values.

A

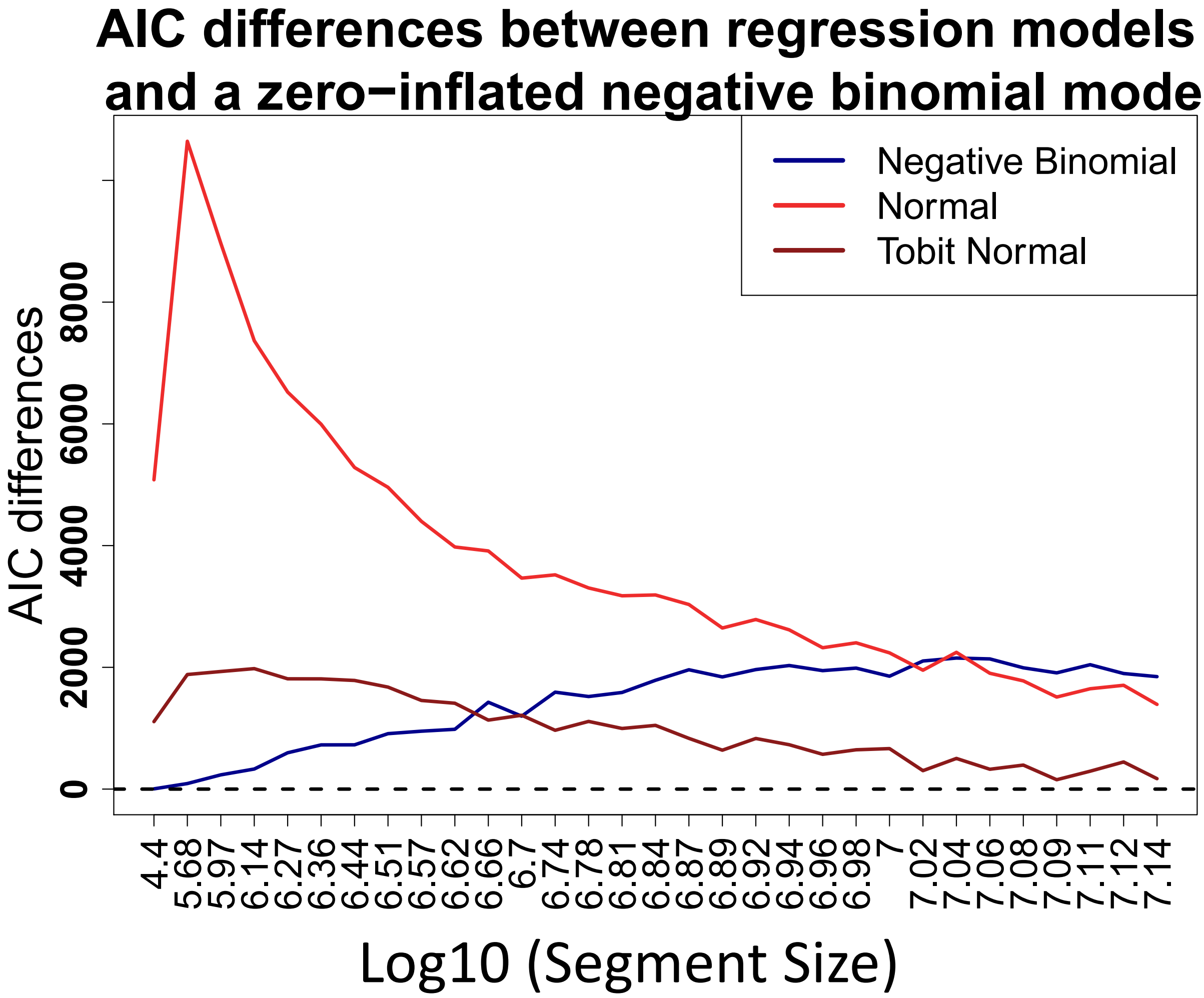

AIC differences between regression models and a zero−inflated negative binomial model
Negative Binomial
Normal
Tobit Normal
AIC differences
0
2000
4000
6000
8000
4.4
5.68
5.97
6.14
6.27
6.36
6.44
6.51
6.57
6.62
6.66
6.7
6.74
6.78
6.81
6.84
6.87
6.89
6.92
6.94
6.96
6.98
7
7.02
7.04
7.06
7.08
7.09
7.11
7.12
7.14
Log10 (Segment Size)

B

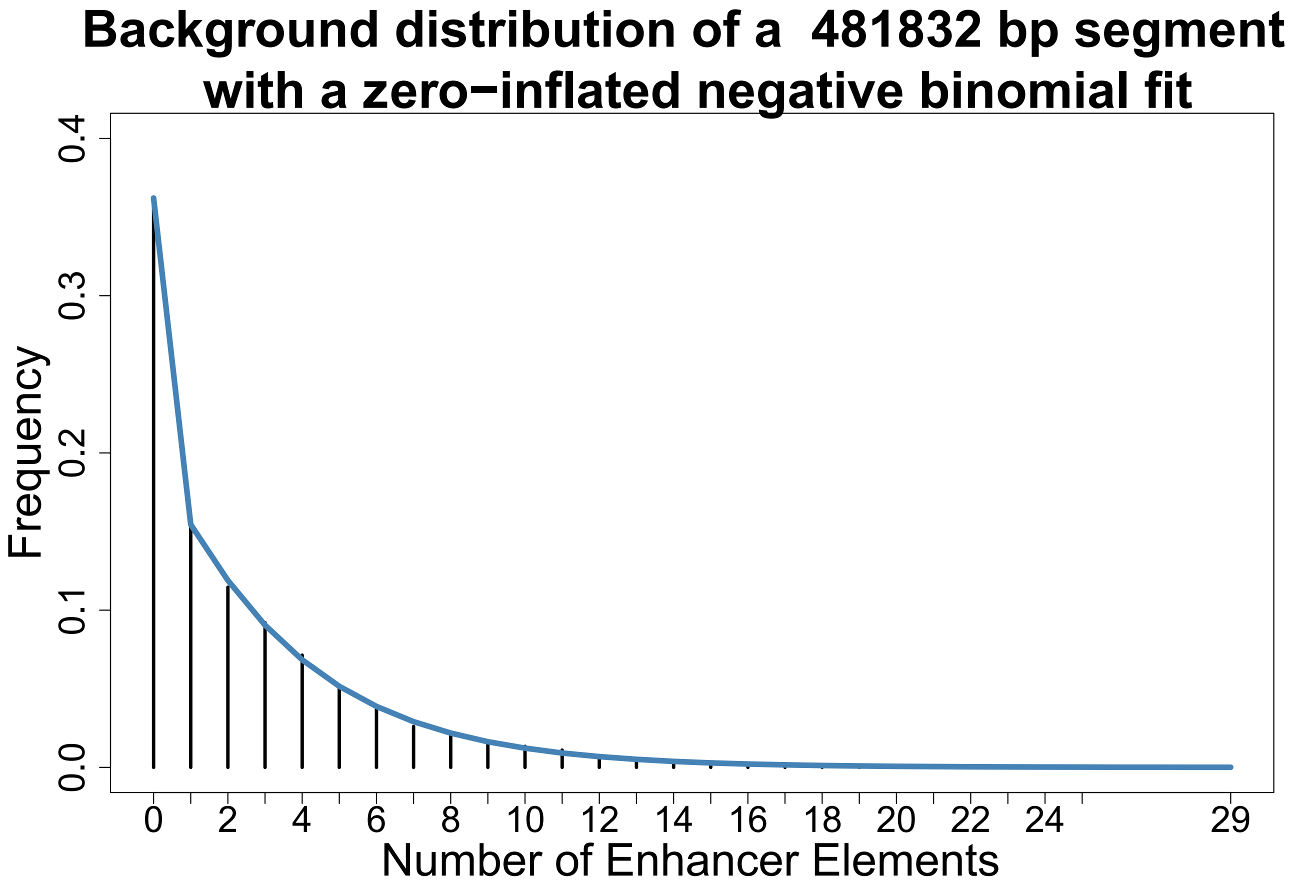

Background distribution of a 481832 bp segment with a zero−inflated negative binomial fit
Frequency
0.0
0.1
0.2
0.3
0.4
0
2
4
6
8
10
12
14
16
18
20
22
24
29
Number of Enhancer Elements

Figure S2. Choosing a background model for the annotation of segments.

A: Different models were fitted for segments of different lengths. The Akaike Information Criterion was used to determine better model fits. AIC differences between the models depicted and a zero-inflated negative binomial model are provided. In all cases the zero-inflated negative binomial model exhibited a superior fit. All models were fit in R with the fitdist() command from the fitdistrplus R package.

B: Example of a zero-inflated negative binomial fit. Segments of 481832 base pairs were shuffled and the number of enhancer elements were counted at each iteration. The blue line depicts a zero-inflated negative binomial distribution fit.

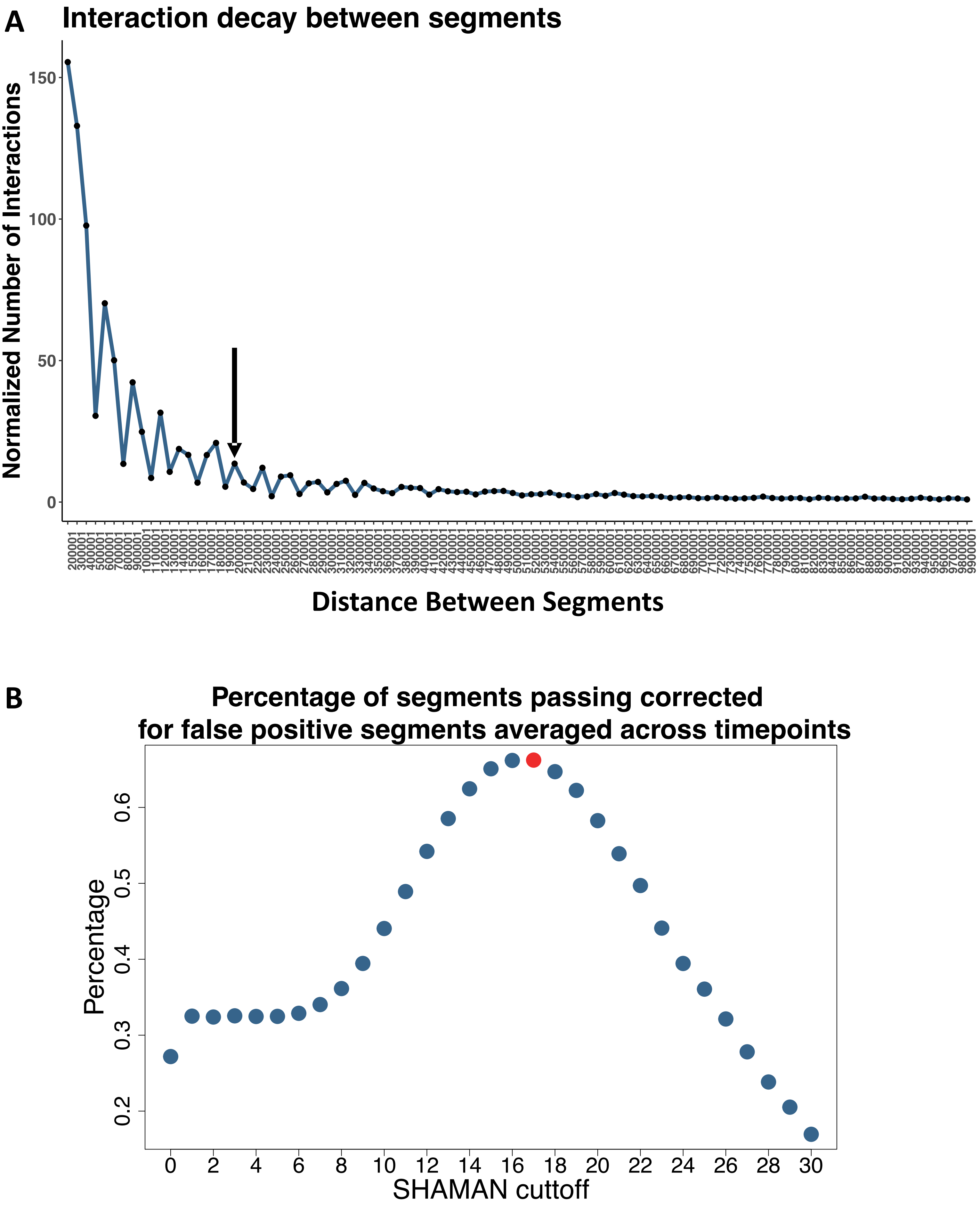

A
Interaction decay between segments
Normalized Number of Interactions
150
100
50
0
Distance Between Segments
B
Percentage of segments passing corrected
for false positive segments averaged across timepoints
Percentage
0.2
0.3
0.4
0.5
0.6
0 2 4 6 8 10 12 14 16 18 20 22 24 26 28 30
SHAMAN cuttoff

Figure S3. Choosing SHAMAN cutoffs for the identification of putative condensates.

A: SHAMAN contacts decay over distance. The number of SHAMAN interactions between randomly picked segments were isolated and scaled for the size of the segments picked. The distance between the segment was also reported. Distances between segments were binned and the average score for each bin was calculated. A limit of 2Mb was imposed as beyond this point, SHAMAN interactions were sparse.

B**:** Choosing an appropriate SHAMAN threshold for the identification of putative transcriptional condensates (PTCs). PTCs were isolated for different SHAMAN cutoffs. Afterwards, segments were randomly generated and were annotated for enhancers. Different SHAMAN cutoffs were once again tried for the set of randomized segments and false-positive PTCs were isolated. The number of segments within false-positive PTCs was collected for each cutoff and averaged across timepoints 0d,1d and 7d. A score was calculated for each SHAMAN cutoff as:

Percentage of actual segments within PTCs passing – Percentage of randomized segments within false-positives PTCs passing

A cutoff of 17 was used, as it seemed to maximize the above score.

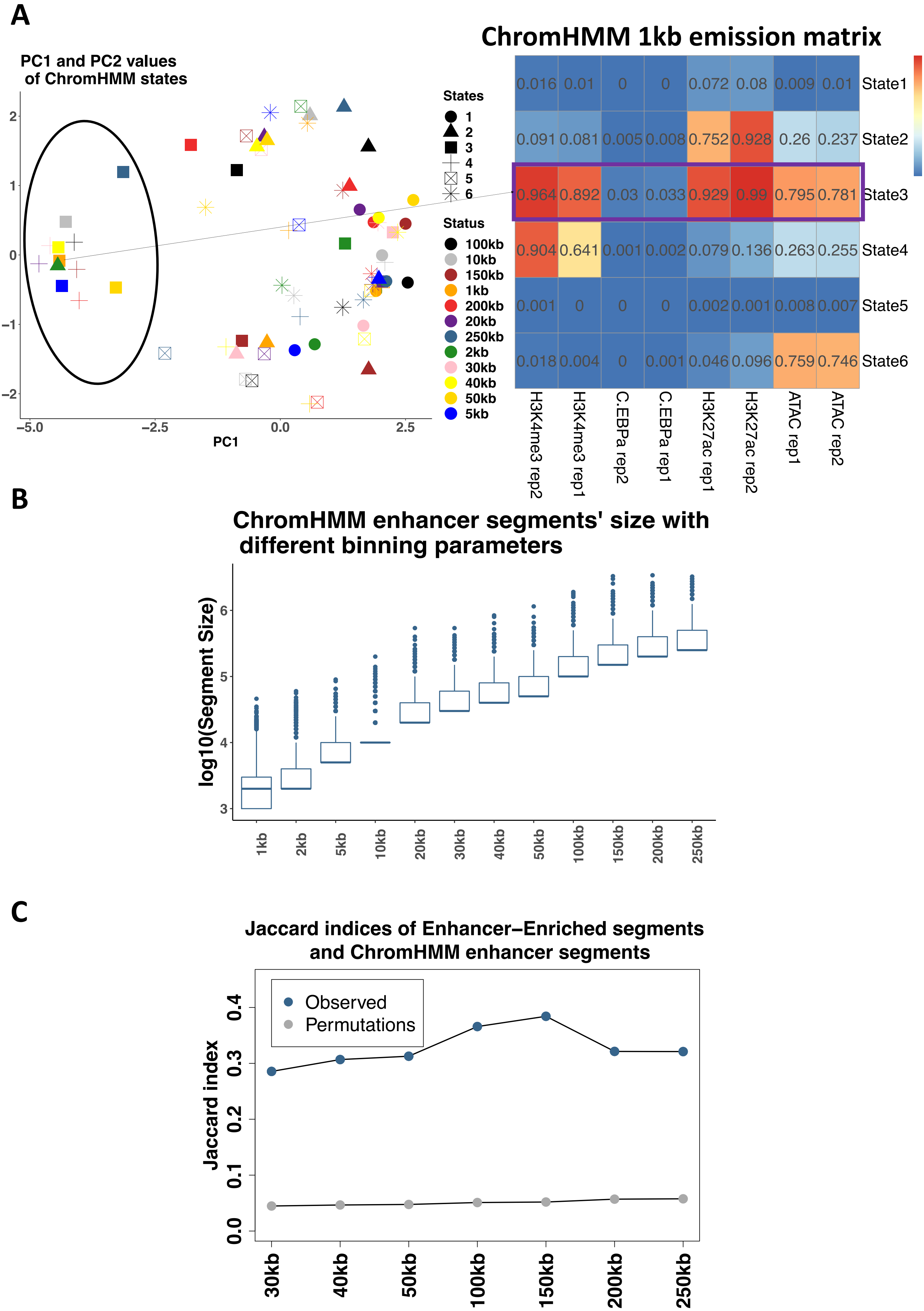
A
PC1 and PC2 values of ChromHMM states
PC1
PC2
−5.0
−2.5
0.0
2.5
−2
−1
0
1
2
States
1
2
3
4
5
6
Status
100kb
10kb
150kb
1kb
200kb
20kb
250kb
2kb
30kb
40kb
50kb
5kb
ChromHMM 1kb emission matrix
0.016 0.01 0 0 0.072 0.08 0.009 0.01 State1
0.091 0.081 0.005 0.008 0.752 0.928 0.26 0.237 State2
0.964 0.892 0.03 0.033 0.929 0.99 0.795 0.781 State3
0.904 0.641 0.001 0.002 0.079 0.136 0.263 0.255 State4
0.001 0 0 0 0.002 0.001 0.008 0.007 State5
0.018 0.004 0 0.001 0.046 0.096 0.759 0.746 State6
H3K4me3 rep2
H3K4me3 rep1
C.EBPa rep2
C.EBPa rep1
H3K27ac rep1
H3K27ac rep2
ATAC rep1
ATAC rep2
0.8
0.6
0.4
0.2
B
ChromHMM enhancer segments' size with different binning parameters
log10(Segment Size)
3
4
5
6
1kb
2kb
5kb
10kb
20kb
30kb
40kb
50kb
100kb
150kb
200kb
250kb
C
Jaccard indices of Enhancer−Enriched segments and ChromHMM enhancer segments
Jaccard index
0.0
0.1
0.2
0.3
0.4
Observed
Permutations
30kb
40kb
50kb
100kb
150kb
200kb
250kb

Figure S4. Benchmark of ChromHMM technical details.

A: ChromHMM runs with different binning parameters and 6 states. Emission states of each run were collected and analyzed via a PCA analysis. The first two principal components were plotted. States encircled represent enhancer-related states associated with each run. An example of this enhancer-related state can be seen on the right.

B: Size distributions of ChromHMM enhancer segment lengths.

C: Jaccard Indices of Enhancer – Enriched segments and ChromHMM enhancer segments. The jaccard index for each overlap was calculated using bedtools jaccard. Afterwards, ChromHMM enhancer segments were randomly shuffled 1000 times using bedtools shuffle and the jaccard score between ChromHMM enhancer segments and Enhancer – Enriched segments was calculated. The average jaccard index of the 1000 permutations is reported.

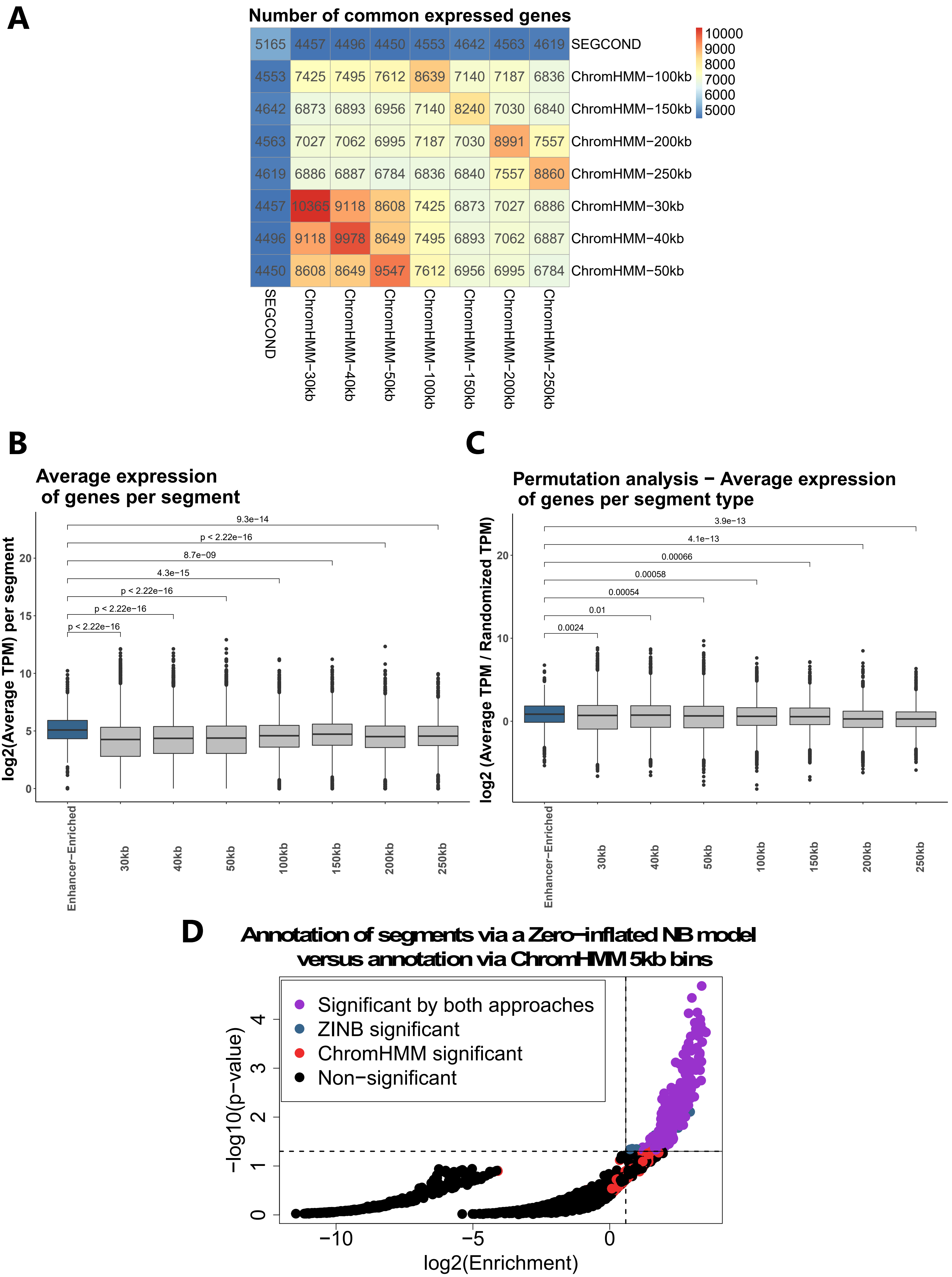
A
Number of common expressed genes
5165 4457 4496 4450 4553 4642 4563 4619 SEGCOND
4553 7425 7495 7612 8639 7140 7187 6836 ChromHMM−100kb
4642 6873 6893 6956 7140 8240 7030 6840 ChromHMM−150kb
4563 7027 7062 6995 7187 7030 8991 7557 ChromHMM−200kb
4619 6886 6887 6784 6836 6840 7557 8860 ChromHMM−250kb
4457 10365 9118 8608 7425 6873 7027 6886 ChromHMM−30kb
4496 9118 9978 8649 7495 6893 7062 6887 ChromHMM−40kb
4450 8608 8649 9547 7612 6956 6995 6784 ChromHMM−50kb
SEGCOND
ChromHMM−30kb
ChromHMM−40kb
ChromHMM−50kb
ChromHMM−100kb
ChromHMM−150kb
ChromHMM−200kb
ChromHMM−250kb
10000
9000
8000
7000
6000
5000
B
Average expression of genes per segment
log2(Average TPM) per segment
9.3e−14
p < 2.22e−16
8.7e−09
4.3e−15
p < 2.22e−16
p < 2.22e−16
p < 2.22e−16
Enhancer−Enriched
30kb
40kb
50kb
100kb
150kb
200kb
250kb
C
Permutation analysis − Average expression of genes per segment type
log2 (Average TPM / Randomized TPM)
3.9e−13
4.1e−13
0.00066
0.00058
0.00054
0.01
0.0024
Enhancer−Enriched
30kb
40kb
50kb
100kb
150kb
200kb
250kb
D
Annotation of segments via a Zero−inflated NB model versus annotation via ChromHMM 5kb bins
Significant by both approaches
ZINB significant
ChromHMM significant
Non−significant
−log10(p−value)
log2(Enrichment)

Fig S5. Benchmark of segmentation and annotation steps versus ChromHMM.

A: Number of common expressed genes between ChromHMM enhancer segments and "Enhancer – Enriched" segments. Pseudogenes and genes with a TPM value of zero were excluded.

B: Average expression of genes per segment of different types. wilcox.test() p-values are also provided.

C: Average expression enrichment scores for Enhancer – Enriched segments and ChromHMM enhancer segments. A permutation analysis was used to assess whether Enhancer – Enriched segments tend to harbor higher expressed genes than ChromHMM enhancer segments.

D: Annotation of segments via a ChromHMM based method and a zero-inflated negative binomial model. Each dot represents a single segment.

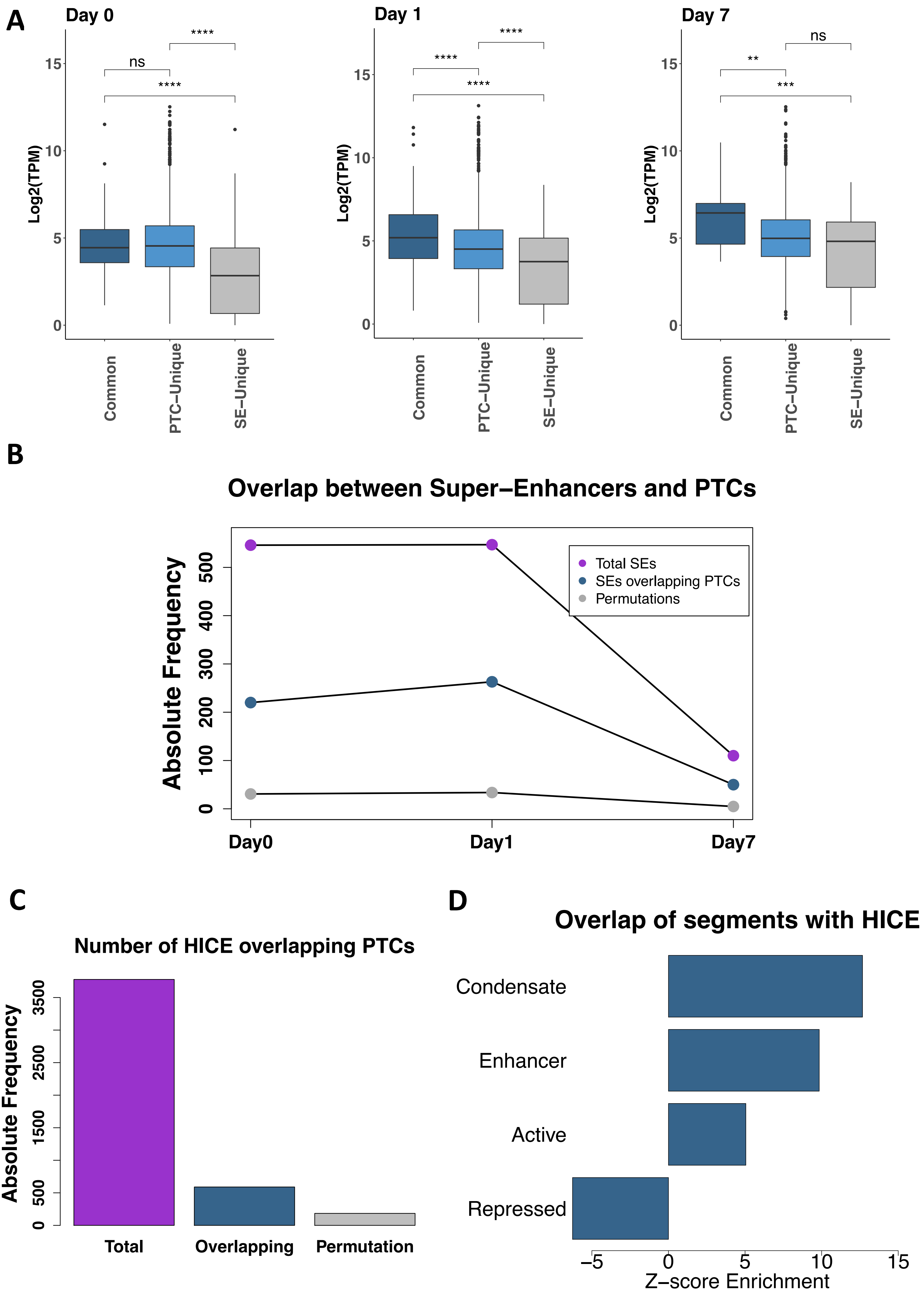
A
Day 0
Day 1
Day 7
Log2(TPM)
ns
****
**
***
Common
PTC–Unique
SE–Unique
B
Overlap between Super–Enhancers and PTCs
Absolute Frequency
Total SEs
SEs overlapping PTCs
Permutations
Day0
Day1
Day7
C
Number of HICE overlapping PTCs
Absolute Frequency
Total
Overlapping
Permutation
D
Overlap of segments with HICE
Condensate
Enhancer
Active
Repressed
Z–score Enrichment

Figure S6. Comparison of SEGCOND output with HICE and Super Enhancers

A: Expression levels of super - enhancer genes, identified on the basis of H3K27ac signal, within and outside PTCs. For each timepoint a set of enhancers was isolated: H3K27ac and ATAC peaks were called using MACS2 (https://github.com/jsh58/MACS) with default parameters. Common H3K27ac and ATAC peaks were kept per time point. The ROSE algorithm was run with default parameters (and the option -t 2.500) and the mentioned peak file per time point. Expression levels of genes common between the two approaches versus Super-Enhancer related genes not within PTCs are compared. Stars denote a wilcox.test() p-value of <0.0001 (****), of <0.001 (***) or of <0.01 (**).

B: Overlap of Super-Enhancers and PTCs. The absolute numbers of Super-Enhancers, and the absolute numbers of Super-Enhancers overlapping a PTC per timepoint are illustrated. Overlaps of at least 1 base pair were calculated using bedtools intersect. A permutation analysis was also performed to evaluate whether the observed overlap is enriched for each timepoint. Genomic coordinates of Super-Enhancers were randomly shuffled using bedtools shuffle for a thousand iterations and the number of overlaps with PTCs was calculated for each iteration. The average number of overlaps of the random iterations is depicted in the plot.

C: Overlap of HICE and PTCs. The absolute numbers of HICE and the absolute numbers of HICE overlapping a PTC are reported. A permutation analysis was carried to determine whether the observed overlap is enriched. Genomic coordinates of HICE were randomly shuffled using bedtools shuffle and the number of overlaps with PTCs was calculated for each iteration. The average number of overlaps of the random iterations is depicted.

D: Z-score enrichments of HICE within different types of segments. SEGCOND was run on a set of datasets derived from BM-hMSC-TER4 cells (https://www.nature.com/articles/s41588-020-0709-z). For the segmentation step binding of MED1, H3K27ac, and C/EBPb alongside DNAse-seq were used (MED1: GSM3100961, GSM3100962, H3K27ac: GSM3100981, GSM3100982, C/EBPb : GSM4340689, GSM4340690, DNAse-seq: GSM3100941, GSM3100942 and Hi-C for the Hi-C integration step: GSM4340725, GSM4340726). Segmentation was performed with the same parameters as the BLaER derived datasets. The overlap between the resulting segments and HICE was calculated with bedtools intersect. To assess statistically the overlap between HICE and each type of segment, a permutation scheme was employed. Segments were shuffled randomly 1000 times using bedtools intersect and the overlap of randomized segments and HICE was calculated. A Z-score was calculated as:

(# Of True Overlap Events - Average # of Randomized Overlap Events) / (Standard Deviation of Randomized Overlap Events)
